# ‘Clinical Is the Pinnacle’: Nurse Academics' Perspectives and Opinions of Their Students Undertaking Mental Health Clinical Placements

**DOI:** 10.1111/inm.13453

**Published:** 2024-10-11

**Authors:** Lorna Moxham, Kim Foster, Brenda Happell, Richard Lakeman, Michael Hazelton, John Hurley

**Affiliations:** ^1^ Faculty of Science, Medicine and Health University of Wollongong Wollongong New South Wales Australia; ^2^ Australian Catholic University Melbourne Victoria Australia; ^3^ Faculty of Health Southern Cross University Lismore New South Wales Australia; ^4^ School of Nursing and Midwifery University College Cork Cork Ireland; ^5^ School of Nursing and Midwifery Edith Cowan University Perth Western Australia Australia; ^6^ University of Newcastle Newcastle New South Wales Australia

**Keywords:** clinical placement, mental health nurse academics, mental health nursing, preregistration nursing curriculum

## Abstract

Clinical placements form an integral and important part of preregistration nursing student learning. The theory–practice gap has been identified as problematic, with clinical experience being a key strategy to address this. Despite this, the perceptions of nurse academics teaching preregistration mental health nursing regarding clinical placements have not been widely explored. To garner perspectives and experiences of mental health clinical placements from nurse academics teaching mental health nursing to preregistration nursing students. A descriptive qualitative study involving 19 nurse academics from 13 metropolitan and regional Australian universities, who were involved in the design and delivery of preregistration mental health nursing content. Data were analysed thematically. The study adhered to the Standards for Reporting Qualitative Research (SRQR). Participants reported that inappropriate clinical placements generate negative student experiences. Furthermore, mental health placements in nonspecialist settings such as medical‐surgical or aged care compromised student learning and posed a barrier to linking theory to practice. Increasing meaningful and appropriate mental health clinical placements in nurse education requires investment and support from multiple stakeholders. Nurse academics are crucial stakeholders in terms of understanding the impact of mental health clinical placements. Appropriate mental health clinical placements are central to effective comprehensive nurse education. Academics teaching mental health in preregistration curricula are significant stakeholders, and their informed perceptions are central to compel change.

## 1 Introduction

It is not uncommon to hear preregistration nursing students say that for them ‘clinical is the pinnacle’. This colloquial saying not only demonstrates the importance of clinical placement in their minds, but also reflects students' views that practical experience is an exciting and integral part of their nurse education. These clinical placements, also known as Work Integrated Learning or WIL, ‘refers to the provision of work‐based experiences within a curriculum that provide opportunities for students to apply theoretical learnings in practice’ (Usher et al. 2022, 155). Clinical placements enable students to experience what it is like to work as a health professional in real‐life workplace settings and are an integral part of learning how to be a registered nurse (O'Brien, McNeil, and Dawson 2019). In this regard, it is important that clinical placements afford preregistration nursing students the opportunity to experience clinical settings which include exposure to excellent mental health nursing practice.

## 2 Background

A clinical placement is an arrangement in which a student is engaged in learning, observation and supervised practice in a healthcare practice environment. The objective of a clinical placement was to provide students with an opportunity to apply what they have learned in their programme to a clinical setting. Students utilise their theoretical knowledge in a supernumerary training environment to broaden their nursing understanding and application. This is achieved by gaining hands‐on experience, learning new skills and cultivating understanding of different areas of practice (O'Brien, McNeil, and Dawson 2019). While on clinical placement students learn much more than psychomotor task application. They also more fully comprehend scope of practice requirements, the need for teamwork and effective communication, and can appreciate the need to prioritise and manage time and how to tackle complex situations (Lee, Clarke, and Carson 2018). During clinical placements, Patel and Metersky (2022) assert that reflective practice is a cognitive skill that demands conscious effort to look at a situation with an awareness of own beliefs, values and practice enabling nurses to learn from experiences, incorporate that learning in improving patient care outcomes. It also leads to ‘knowledge development in nursing’ (p. 180). Furthermore, attending clinical placements allows students to gain a sense of belonging (Levett‐Jones et al. 2008), develop clinical reasoning (Price 2019) and obtain genuine insights into the diverse nursing career pathways available to them when they graduate (Foster et al. 2021).

In Australia, where this study is situated, students enrolled in a preregistration comprehensive nursing degree at a higher education institution typically complete 16–24 subjects and have a minimum of 800 mandated hours of clinical placement. Despite the intent to offer students a variety of placements so that the above learning objectives can be achieved, not all students get to attend all nursing practice areas. Some universities, for instance, do not send their students on a mental health clinical placement. At other universities, students undertake a ‘mental health’ placement in a nonmental health setting such as medical‐surgical or aged care, reflecting the view that people with mental health conditions are in every health setting (Happell and Platania‐Phung 2012) and that essential nursing skills can be gained across a broad range of settings.

The issue of mental health clinical placements for nursing students is an international concern. While there have been a number of studies exploring nursing students' experiences of mental health clinical placements (e.g., Foster et al. 2021; Happell et al. 2015; Hall et al. 2021; Goman et al. 2020), there is little prior evidence on the perceptions of nurse academics regarding mental health clinical placements.

## 3 Aim

The aim of this research therefore was to garner perspectives and experiences of mental health clinical placements from nurse academics teaching mental health nursing to preregistration nursing students.

## 4 Methods

### 4.1 Design

The research aim was explored using a descriptive qualitative design (DQD) (Doyle et al. 2020) which is considered a useful means of exploring perceptions and opinions of target populations. Given research in this area is minimal, DQD is appropriate. The results being reported in this paper are part of a larger mixed‐methods study of mental health nurse academics in Australia which explored perceptions regarding the adequacy of the content and process of preregistration programmes to prepare nursing graduates to work in mental health settings and with people experiencing mental health problems. The study adhered to the Standards for Reporting Qualitative Research (SRQR) (O'Brien et al. 2014).

### 4.2 Sample

Purposive sampling was undertaken for this research, targeting mental health nurse academics teaching into preregistration nursing curricula at universities across Australia.

### 4.3 Data Collection and Participant Characteristics

Prior to data collection, the Human Research Ethics Committee (HREC) from the relevant organisation provided approval for this study (2023/05). All participants gave informed written consent prior to the participation. The research began by sending an online Qualtrics survey to all Australian universities who held membership of the Council of Deans of Nursing and Midwifery. The survey included an invitation for staff to participate in an interview. After completing the survey, 19 academics representing 13 universities agreed to be interviewed for the qualitative component of the overarch project. The 19 academics were located in metropolitan (*n* = 6) and regional (*n* = 7) locations. In the Australian context, most metropolitan universities also have regional campus’s and course coordinators oversee all students irrespective of campus location. At times this could mean coordinating a course with over 1,000 students spread across wide geographic areas. Clinical placement teams are though, often located centrally in an attempt to procure more placements. The interviews were held between May and June 2023 and conducted by members of the authorship team, all qualified and experienced qualitative researchers. An interview guide provided research focused questions and was conducted online to overcome large geographical limitations related to the tyranny of distance in Australia. Interviews were audio‐recorded and lasted between 40 and 60 min. Transcript accuracy was cross‐checked against recordings by two members of the author team.

Nine participants identified as female with 10 identifying as male. All participants identified as possessing specialist mental health qualifications. They had experience in mental health academia ranging from two to approximately 20 years. During the interviews, participants were asked their perspective and experiences of mental health clinical placements. This topic is the focus of the current paper.

### 4.4 Data Analysis

Extensive data were generated through the questions regarding clinical placements. Data were analysed using Braun and Clarke's approach to descriptive thematic analysis. Using this approach, as well as entering data into NVivo to assist with management and organisation, facilitated the identification and analysis of patterns or themes in the data set (Braun and Clarke 2012). The data analytic approach is a phased process that facilitated the analysis and aided the research team to identify important aspects of a thematic analysis. Preliminary thematic nodes which were a collection of references from the files about a specific theme, topic, concept, idea, or experience, were identified. The nodes were organised into themes by analysing the meaning of words and sentence structure. Independent analysis was undertaken by three members of the research team, all experienced nurse academics and researchers. Differences were deliberated until consensus was achieved with the agreed thematic results being reviewed and approved by all members of the research team.

## 5 Results

Three superordinate themes were identified, as indicated in bold font. Findings from analysis suggest that *Student experiences influence intended area of practice*, that the *Appropriateness of mental health placements* used are nonspecific to mental health nursing and that learning was compromised as a result and that *Placement timing and length* is not conducive to linking theory to practice.

### 5.1 Student Experiences Influence Intended Area of Practice

Participants suggested that depending on several factors such as the health service or organisation's culture and the engagement of clinical facilitators, students could have either a positive or negative experience of placements. This experience was then thought to be influential for future areas of practice. A participant said ‘if they have a good placement they consider mental health nursing as an option and the opposite happens if their placement is not so good’. Participants, spoke about their awareness of the importance of placements in influencing students' views on mental health with statements such as, ‘it's the face‐to‐face experiential placement that will shift people's thinking and go. My Gosh! This is it? This is where I want to be’.

Experiences were broadly spoken about as either positive or negative and the subsequent influence this had on future intention to practice with the specialty area. Previous research related to students' clinical placements, reports on positive aspects of their experience. Participants in this study also described positive experiences, stating that these were influential when students considered mental health nursing postdegree completion. One participant described how his students ‘really enjoyed the placement, that it was very interesting, and made them interested in mental health nursing as a clinical option, when they had never thought about that before’.

However, participants also openly and honestly disclosed that some students returned from their mental health clinical placement and described it as a negative experience. One participant described how excited they were initially when students went on mental health clinical placement, ‘I was so excited about what this placement might mean’. However, these expectations were dashed with the participant talking about how they felt when a student subsequently described her placement experience when she returned to university. The participant recalled that the student said it was ‘devastating for me as it was just one of the worst placements I've ever done’.

The negative student placement experiences reported by participants resulted in students telling them that they had encountered less than positive interactions with staff (‘not students again, we don't have time for you’), not being allowed to do anything while on placement (‘don't leave the nurses station’) and organisational culture that was not optimal (‘the environment was toxic’). Participants described that these negative experiences had been influential in that it made students feel like ‘mental health nursing was not for them’.

### 5.2 Appropriateness of Mental Health Placements

Participants revealed that the type of clinical placements students were sent to for their mental health experience varied widely. Apparently, often due to purported lack of mental health placement opportunities, students undertook their mental health clinical placements in non‐mental health settings. These were reported to be in ‘childcare centres, doctors' surgeries, medical wards, and aged care facilities’. The rationale given by university's for sending students on a mental health clinical placement in areas that participants said were ‘really inappropriate’ was ‘mental health is everywhere’. Despite this rationale, participants felt that ‘placements are a numbers game’ and difficulty acquiring mental health clinical placements in adequate numbers was consistently reported, ‘It is heavily dependent on where a placement arises. It varies hugely’.

Some participants described students being sent on mental health clinical placements to ‘GP clinics and aged care facilities with dementia and behavioural units’ and spoke about ‘the substitution of actual mental health placements with placements in older, like geriatric placements, and those sorts of things’. They did not think these ‘substitute placements’ were appropriate to learning about mental health nursing.

Of concern was that mental health clinical placements were either not mandated at some university's or not offered at all, meaning that a considerable number of students received no mental health learning in practice, during their preregistration degree programme despite the outcomes intending ‘comprehensive training’. This is illustrated by the following participant when they said; ‘50% of the students were going on mental health specific placements, and others didn't know whether they were ever going to get a mental health placement, so they'd be sent on a general placement. Training is oriented toward general nursing. It isn't comprehensive at all’.

### 5.3 Placement Timing and Length

Participants considered that the timing of placements in terms of proximity to theoretical instruction was important in terms of students connecting learning. Often, student learning in practice occurred either before, or a long time after, mental health theoretical instruction. Participants described:

there is still that loss of connection between that (theory) and requirements for students to engage in a clinical placement *and that* we then can't consolidate the theoretical concepts in the programme in the clinical environment.

Over the course of their degree students undertake 800 h of mandatory clinical placement. Of these 800 h, participants identified that the average mental health clinical placement at their university was ~80 h (usually over a 2‐week block) but that even that was said to be diminishing;

we went from like four weeks, then we went down to three weeks, and now they're talking about two weeks and simulations and so on as replicating it and even trying the old argument that students don't actually need mental placements at all, we can just do it all by simulation.

Unfortunately, some participants described students not having a mental health clinical placement at all, ‘we do not have a compulsory clinical placement attached to this programme which I think is very sad as mental health is a part of life, it's a holistic part of care’.

Sixteen out of 19 (84%) of participants suggested that preregistration clinical placements to enable the development of mental health nursing skills were inadequate with participants lamenting that,

there doesn't seem to be a willingness for the university sector or the clinical provision providers out there to actually bridge that gap simply. So we struggle in preparation.

## 6 Discussion

This paper provides an important narrative on a primary study regarding academics' perspectives on mental health clinical placements and reinforces some of the prior evidence on these specialist placements. Participants described that students could have both positive and negative experiences of mental health placement and that the experience influenced whether or not they considered practising in the area postdegree completion. This is consistent with some prior research, where students have been reported encountering both negative and positive experiences (Zulu, du Plessis, and Koen 2021) and that they influenced practice choice (Rodriguez‐Garcia et al. 2021; Tapsell et al. 2021; Slemon et al. 2020). Not only do clinical placements provide an authentic learning experience of day‐to‐day clinical practice, but also they also provide students with genuine opportunities to learn from registered nurses' clinical reasoning (Fedele 2023; Partington, Brook, and McKeown 2024). Learning in practice through clinical placements is a critical component of nurse education (Cant et al. 2021). As Hall, McFarlane, and Mulholland (2013) suggest, many placements are considered positive, but a negative experience can have significant impact on students, clinicians and clinical sites resulting in an agency's reluctance to accept nursing students or an educator's reluctance to send students to specific placement sites. With regard to placement preference, Machindo et al. (2019) explicated that students prefer acute and critical care experiential learning opportunities. Research related to the quality of student mental health clinical placement experiences and the effects these have on students have been consistently raised by researchers such as Happell et al. (2015), Stuhlmiller and Tolchard (2019), Buchanan (2021) and Moxham et al. (2023). Such is the importance of clinical placements, the Australian Council of Deans of Nursing and Midwifery (CDNM) called for a systematic approach to addressing the variance in quality of student learning experiences (Fedele 2023). A study undertaken by the National Placement Evaluation Centre (NPEC) has now been established which purports to ‘undertake nursing and midwifery student placement feedback in order for stakeholders to provide improvements in clinical education’ (Ryan, Cooper, and Cant 2023, 31). What is known already, and reinforced through the results of this current study, is that student experience of clinical placements has a formative influence on graduates' intentions to practice in a particular specialty.

The current study also found that some universities were reported by participants as holding the view that many ‘alternative’ types of placements were considered relevant for mental health clinical experience. The rationale was that all healthcare settings have people with mental health conditions. There clearly remains some contention about what counts as an appropriate mental health placement. It is most unlikely that a medical or surgical placement might occur in a mental health centre, even though people accessing mental health services have physical illnesses and injuries at a rate significantly higher than the general population (Morgan, Waterreus, and Ambrosi 2020). Placing students in a nonmental health setting could be seen as an easier option by universities who are struggling to find specialist mental health placements. However, mental health care is a specialist field of health. A nonspecialist setting does not provide students with access to mental health consumers receiving specialist mental health care, nor to specialist staff who can provide role modelling and mentoring in mental health‐specific skills, nor does it provide experience of mental health models of care (Happell and Platania‐Phung 2012). These are important aspects of learning about specific mental health nursing skills that are needed by students.

In line with mental health issues being increasingly prevalent within the community (Halcomb et al. 2018) and identified mental health‐related global issues and solutions by international bodies such as the World Health Organization (2022), mental health is a discreet area recognised in Australia by its own political Ministerial portfolios (e.g., Minister for Mental Health), a specific National Mental Health and Wellbeing Roadmap (Council of Australian Governments 2012) and national grant funding schemes (e.g., Million Minds Mental Health Research Mission). Mental health nursing is a specialty field and was recognised as such for decades in Australia with specialist registration (Foster and Hurley 2024) and is still recognised as a specialty in countries such as the UK and Republic of Ireland (Lakeman et al. 2023). Indeed, the World Health Organization (2022) has recently noted that a competent specialist workforce forms the backbone of any mental health system.

In this regard, mental health nursing clinical placements need to occur in mental health settings with mental health nurses as facilitators (Perlman et al. 2020). Placement environments need to be conducive to enable students to deeply engage in the skills, knowledge and attitudes required to ensure people with a lived experience receive quality care (Perlman et al. 2017). Research exploring the impact of nonmental health settings on nursing students' attitudes to mental health suggests a far lesser impact on attitudes to, and interest in, mental health nursing than when placements are undertaken in specialist mental health settings (Barrett and Jackson 2013; Happell and Platania‐Phung 2012).

In Australia, preregistration nursing students must complete a minimum of 800 h of clinical placement throughout their course (Australian Nursing and Midwifery Council 2016) Participants in this research indicated that most students undertook only one mental health subject and only had 80 h clinical placement (out of 800). Several participants described how some students had no mental health clinical placement at all. With one in five Australians reported to experience a mental illness (Moxham et al. 2023), it is difficult to see how appropriate care can be provided if clinical education in this area is lacking.

The goal of preregistration learning is the development of safe and competent clinical practice (Stanley, Gale, and Mossburg 2023). Therefore, theory and practice are the essential components of nursing education. Theoretical knowledge should underpin practice, while the practice environment provides the setting for theoretical knowledge to be applied (Saifan et al. 2021). The theory–practice gap is a reverberative theme and generative metaphor in nursing education (Salifu et al. 2018), and as long ago as the 1980s the theory–practice gap was described as occurring when theory is not translated into practice (Greenway, Butt, and Walthall 2019). The disconnect occurs when practitioners, such as student nurses, struggle to integrate knowledge learned in an academic environment with real‐world clinical practice (Zieber and Wojtowicz 2019). The theory–practice gap is thought to be able to be overcome by strategies, one of which is the provision of opportunities for experiential learning—vis a vis—clinical placement (Berndtsson, Dahlborg, and Pennbrant 2020).

A principle in Gestalt psychology reminds us that humans perceive elements that are close together as more related than ones farther apart. Situating clinical placements in close proximity to theoretical learning facilitates students' ability to transfer theory into clinical practice. The theory practice gap is best bridged by consolidating theoretical learning in practice in close proximity to expert practitioners. The timing of clinical placements in relation to theoretical instruction can have an impact on student learning. The research from the present study suggests the need for consistent clinical placements, supportive clinical learning environments, and effective coaching by clinical educators to positively affect student outcomes. Continuous placements in the same clinical setting have the potential to offer greater opportunities for learning and early professional socialisation, as well as a more integrated approach to applying classroom theory into practice (Tanda and Denham 2009). However, more research is needed to determine optimal staff–student ratios, clinical hours and clinical sequencing in preregistration nursing education (Romansky et al. 2017; Sheehan and Wilkinson 2018).

### 6.1 Limitations and Strengths

This study was undertaken in one country with a purposive group of university academics, and as such, the findings may not be transferable to all other contexts. As with all research, participants may not be truthful when answering questions or they may have given socially desirable responses and the choice and wording of questions may have influenced the descriptive findings. Strengths of the study included the expertise and experience of the collegiate professoriate who designed the project and consistently engaged in reflexivity. The.

## 7 Conclusion

Despite clinical learning being fundamental to preregistration nursing students' mental health educational journey, the research reported in this paper suggests that mental health clinical placements appear to be occurring in inappropriate settings, for a relatively shorter period in comparison with other practice areas and are purported in some cases to be less than positive experiences. The mental health of Australia and indeed citizens worldwide is declining. People with mental ill health are just as deserving of high‐quality clinical care as people with every other illness, injury and/or disease. This means that clinicians need to have the necessary skills and knowledge to provide this care. This can be achieved through sufficient theoretical education and adequate and appropriate learning in specialist mental health clinical placements. Nurse academics, such as those who participated in this research, have a crucial role as advocates for the provision of appropriate and timely clinical placements through curriculum transformation. The million‐dollar question is: When they speak, will they be heard in the polyphony of voices?

## 8 Relevance for Clinical Practice

This study provides evidence for educators coordinating clinical placements about the specialty nature of mental health practice learning. The study's insights into nurse academics perceptions of speciality clinical experiences in mental health may help inform future practice learning requirements. Mental health clinical placements were shaped by *Student experiences*, the *Appropriateness of mental health placements* and *Placement Timing*. An understanding of the barriers facilitating effective mental health clinical placements, and the importance of specialised mental health placements, can inform nursing academics preparation, engagement and organisation of clinical placements for preregistration nursing students.

## Author Contributions

**Lorna Moxham:** conceptualisation, methodology, software, data curation, formal analysis, writing – original draft preparation, reviewing and editing. **Kim Foster:** conceptualisation, methodology, software, data curation, formal analysis, writing – original draft preparation, reviewing and editing. **Brenda Happell:** conceptualisation, methodology, software, data curation, formal analysis, writing – original draft preparation, reviewing and editing. **Richard Lakeman:** conceptualisation, methodology, software, data curation, formal analysis, writing – original draft preparation, reviewing and editing. **Michael Hazelton:** conceptualisation, methodology, software, data curation, formal analysis, writing – original draft preparation, reviewing and editing. **John Hurley:** conceptualisation, methodology, software, data curation, formal analysis, writing – original draft preparation, reviewing and editing.

## Ethics Statement

Ethical approval was obtained from the relevant institutional ethical board (approval no: 2023/015).

## Conflicts of Interest

The authors declare no conflicts of interest.

## Data Availability

The data that support the findings of this study are available from the corresponding author upon reasonable request.
